# Real-time changepoint detection in a nonlinear expectile model

**DOI:** 10.1007/s00184-023-00904-6

**Published:** 2023-03-30

**Authors:** Gabriela Ciuperca, Matúš Maciak, Michal Pešta

**Affiliations:** 1 Institut Camille Jordan, Université Lyon 1, 43 blvd du 11 Novembre 1918, Lyon, 69622 France; 2 Faculty of Mathematics and Physics, Department of Probability and Mathematical Statistics, Charles University, Sokolovská 49/83, Prague, 18675 Czech Republic

**Keywords:** Asymmetric least squares, Changepoint test, Conditional expectiles, Online detection, Coherent risk measure

## Abstract

An online changepoint detection procedure based on conditional expectiles is introduced. The key contribution is threefold: nonlinearity of the underlying model improves the overall flexibility while a parametric form of the unknown regression function preserves a simple and straightforward interpretation; The conditional expectiles, well-known in econometrics for being the only coherent and elicitable risk measure, introduce additional robustness—especially with respect to asymmetric error distributions common in various types of data; The proposed statistical test is proved to be consistent and the distribution under the null hypothesis does not depend on the functional form of the underlying model nor the unknown parameters. Empirical properties of the proposed real-time changepoint detection test are investigated in a simulation study and a practical applicability is illustrated using the Covid-19 prevalence data from Prague.

## Introduction

It is a common task—not only in statistics—to provide procedures for detecting and estimating changepoints in all kinds of mathematical and stochastic models. Such procedures are also important from a practical point of view and they may be often crucial in many real life problems. For instance, detecting a changepoint in some data generating model may trigger some model retraining mechanisms or, more frequently, it may govern important decisions effecting specific subjects or even the whole population—such as different pandemic restrictions related to the recent Covid-19 infection spread. On the other hand, the estimation of changepoints may lead to some correction procedures, specific treatment implementations, additional target-specific decisions, or just a deeper understanding of the underlying data generating process.

Considering the basic stochastic principles of the changepoint detection and various estimation methods, two different approaches are usually adopted in practical implementations. If the whole data sample is available at the very beginning of the analysis, the detection algorithm is called an *offline procedure*. If the data arrive in time (usually in an observation-by-observation manner) and the changepoint detection algorithm runs concurrently as new observations appear, such algorithms are referred to as *online procedures*.

In this paper, we focus on the *online regime*, where the proposed changepoint detection algorithm will be applied for a nonlinear parametric regression model. In addition to this nonlinearity, the conditional expectile estimation of the unknown parameters is adopted—similarly as in Newey and Powell (1987) where, however, the authors investigated a simple linear model instead—to have a coherent risk measure while also accounting for possibly asymmetric random error distributions. The changepoint detection itself is performed in terms of a consistent statistical test which is based on an accumulating dataset used in each consecutive step of the proposed *online procedure*.

There is a vast literature available on both—the offline and online changepoint detection strategies considering different models and various technical assumptions. Bearing in mind just the *online procedures*, Nedényi (2018) proposed an online testing approach based on a CUSUM test statistic to detect changes in a parameter of a discrete-time stochastic process. Linear regression models with independent error terms are considered in Chu et al (1996) and Horváth et al (2004), where a standard least squares estimator is employed. Possible detection delays in a sequential changepoint test for a multiple linear regression model are discussed in Aue et al (2009). Linear regression models with dependent observations are investigated in Fremdt (2015) and the online changepoint detection procedures within autoregressive times series are studied, for instance, in Hušková et al (2007). Some generalizations for multivariate cases can be found in Aue et al (2009) or Hoga (2017) and their results are further generalized in Barassi et al (2020) where a semiparametric CUSUM test is proposed to perform the online changepoint detection for various correlation structures of nonlinear multivariate regression models with dynamically evolving volatilities. Nonlinear integer-valued times series are also discussed from this perspective in Lee and Lee (2019). A very nice overall review of the *online procedures* can be also found in Basseville and Nikiforov (1993).

The method presented in this paper advocates the idea of semi-parametric CUSUM approaches in a combination with some robustness with respect to the underlying error terms. Firstly, a nonlinear regression model is assumed to govern the data generating process. Although the underlying regression function is deterministic, it is allowed to be nonlinear with respect to a set of unknown parameters. This introduces a relatively flexible class of possible functions. Second, despite the independent error terms assumed for the proposed online detection regime, there are no restrictive assumptions imposed on the underlying error distribution and, in particular, substantial robustness is achieved with the proposed expectile estimation that also allows for asymmetric and heavy-tailed error distributions. The conditional expectiles define the only coherent and elicitable risk measure (see, for instance, Bellini et al (2018) or Ziegel (2016)) which is particularly important in situations where some risk related assessment is needed. Moreover, despite many similarities with conditional quantiles, the conditional expectiles are well-known to be viable also in situations when the conditional quantiles fail (see Philipps (2022) for a more comprehensive comparison). Third, the proposed test statistic follows, under the null hypothesis of no change, a relatively simple distribution which does not depend on the underlying regression function nor the set of the unknown parameters. Finally, the whole procedure can be implemented in a straightforward way and all necessary calculations performed within the proposed *online regime* can be easily obtained. Thus, the presented real-time changepoint detection method has a great potential for a practical applicability which goes way beyond the Covid-19 example illustrated at the end.

The rest of the paper is structured as follows: The underlying data and the corresponding changepoint model are described in the next section. A real-time changepoint detection in terms of a formal statistical test is introduced in Sect. 3. The asymptotic properties of the proposed test are also detailed there. In Sect. 4, finite sample properties are investigated and the Covid-19 prevalence data from Prague, Czech Republic, are analysed using the proposed methodological framework. Section 5 concludes with some final remarks. All theoretical proofs and further technical details are postponed to the Appendix.

## Asymmetric least squares with changepoint

Let us consider a set of *historical data* denoted as $\{(Y_i, \textbf{X}_i^\top )^\top ;~i = 1, \dots , m\}$ for some deterministic *q*-dimensional vector of explanatory variables $\varvec{X}_i = (X_{i 1}, \dots , X_{i q})^\top$ and some integer $m \in \mathbb {N}$. The data are assumed to follow a general nonlinear parametric regression model

1 $$\begin{aligned} Y_i=f(\textbf{X}_i, {\varvec{\beta }})+\varepsilon _i, \qquad i=1, \dots , m, \end{aligned}$$

where $f(\cdot , {\varvec{\beta }})$ is an explicit function depending on some unknown vector parameter $\varvec{\beta } = (\beta _1, \dots , \beta _p)^\top \in \Gamma \subseteq \mathbb {R}^{p}$ with the true (unknown) value denoted as ${\varvec{\beta }}^0 \in \mathbb {R}^p$. A different approach could consider $\varvec{X}_i$’s as random vectors, however, we concentrate on the fixed design as we want to adopt a robust (i.e., distribution-free) approach with only minimal assumptions being imposed on the underlying data distribution. Nevertheless, with respect to the forthcoming theory, analogous results for the random design can be derived as well (all under some technical assumptions needed for the deterministic convergences to become convergences in probability).

After the *historical data* are observed, another $$T_m \in \mathbb {N}$$ observations are measured instantly for both—the response variable $$ Y_i$$ and the explanatory vector $$\textbf{X}_i \in \varUpsilon \subseteq \mathbb {R}^q$$, both for $$i = m + 1, \dots , m + T_m$$. The underlying model for these new observations—*online data*—is assumed to take an analogous form

2 $$\begin{aligned} Y_i=f(\textbf{X}_i, {\varvec{\beta }}_i)+\varepsilon _i, \qquad i=m+1, \dots , m+T_m, \end{aligned}$$

where the underlying regression functional form remains the same and ${\varvec{\beta }}_i \in \mathbb {R}^p$. For the parameter vectors $\{\varvec{\beta }_i\}_{i = m + 1}^{m + T_m}$ in (2), it is either assumed that their true (unknown) values are all equal to ${\varvec{\beta }}^0$ (thus, there is no changepoint present in the overall combined model (1) and (2)) or, instead, there is some specific index $k_m^0 \in \{1, \dots , T_m - 1\}$ such that ${\varvec{\beta }}_i = {\varvec{\beta }}^0$ for all $i = m + 1, \dots , m + k_m^0$, while ${\varvec{\beta }}_i \ne {\varvec{\beta }}^0$ for $i = m + k_m^0 + 1, \dots , m + T_m$. In such case, there is a changepoint (located at $k_m^0$) present in the model generating the *online data* $\{(Y_i, \textbf{X}_i^\top )^\top ;~i = m + 1, \dots , m + T_m\}$.

The error terms $\{\varepsilon _i\}_{1 \leqslant i \leqslant m+T_m}$ from the overall model (1) and (2) are assumed to be independent and, moreover, they all follow the same distribution. A generic random error term from the underlying distribution is denoted as $\varepsilon$. The idea is to use the *historical data* to estimate the unknown parameter vector ${\varvec{\beta }}\in \mathbb {R}^p$. Later, the *online data*—starting from the observation index $i = m+1$—are measured in real-time while asking a question for each new observation $i \ge m+1$ whether the underlying model remains unchanged (i.e., ${\varvec{\beta }}_i={\varvec{\beta }}^0$) or there is some change detected in terms of the unknown parameter vectors ${\varvec{\beta }}_i \in \mathbb {R}^p$. If there is no changepoint detected for the given *i* then all available observations are used in the next step to ask the same question regarding the new—most recent observation. The whole changepoint detection process stops at the first observation $i \in \{m + 1, \dots , m + T_m\}$ for which there is a statistical evidence that ${\varvec{\beta }}_i \ne {\varvec{\beta }}^0$.

From a formal theoretical point view, at the first step, the *historical data* $\{(Y_i, \textbf{X}_i^\top )^\top :\,i = 1, \dots , m\}$ are used to obtain a conditional expectile estimator for the unknown parameter vector ${\varvec{\beta }}\in \mathbb {R}^p$. In particular, for a given expectile index $\tau \in (0,1)$ the expectile function is defined as

3 $$\begin{aligned} \rho _\tau (x)= \bigg | \tau - \mathbb {I}_{\{x <0\}} \bigg | x^2, \qquad \text {for} \quad x \in \mathbb {R}, \end{aligned}$$

and the corresponding expectile estimator of the unknown (true) parameter vector ${\varvec{\beta }}^0 \in \mathbb {R}^p$ from the model in (1) is defined as

4 $$\begin{aligned} \widehat{\varvec{\beta }}_m\equiv {\mathrm {arg\,min}}_{{\varvec{\beta }}\in \mathbb {R}^p} \sum ^m_{i=1} \rho _\tau \big (Y_i-f(\textbf{X}_i, {\varvec{\beta }})\big ), \end{aligned}$$

where $\widehat{\varvec{\beta }}_m = \big ( \widehat{\beta }_{m 1}, \dots ,\widehat{\beta }_{m p} \big )^\top \in \mathbb {R}^p$. It is straightforward to verify that for $\tau =1/2$ the expectile estimate $\widehat{\varvec{\beta }}_m$ defined by (4) reduces to a standard (nonlinear) least squares (LS) estimator of ${\varvec{\beta }}^0 \in \mathbb {R}^p$. In general, the $\tau ^{th}$ expectile of the given distribution can be interpreted as a hypothetical mean of some other distribution that would be obtained if the values above the expectile in the original distribution would occur $\frac{\tau }{1 - \tau }$ times more frequently. Thus, the choice of $\tau \in (0,1)$ can be also seen in terms of some “exploratory” approach that somehow “balances” the distribution towards the (zero) mean and it provides a useful information about the skewness and possible outlying/extreme observations. Also note, that depending on the choice of the regression function *f*, the minimization problem in (4) may or may not be a convex problem. This restricts the choice of the algorithm used to obtain the final solution. For numerical issues and different techniques for fitting nonlinear models we refer to Chambers (1973). Computational aspects are further discussion in Sect. 4.

In the second step, the expectile estimator $\widehat{\varvec{\beta }}_m$ obtained from the *historical data* $\{(Y_i, \textbf{X}_i^\top )^\top ;~i = 1, \dots , m\}$ is used to perform a real-time changepoint detection in the *online data* $\{(Y_i, \textbf{X}_i^\top )^\top ;~i = m + 1, \dots , m + T_m\}$ in terms of a formal statistical test of the null hypothesis

5 $$\begin{aligned} H_0: \, {\varvec{\beta }}_i= {\varvec{\beta }}^0, \qquad \text {for }\;\; i=m+1, \dots , m+T_m; \end{aligned}$$

against the alternative hypothesis

6 $$\begin{aligned} H_1:&\exists k^0_m \in \{1, \dots , T_m - 1\} \nonumber \\&\text {such that} \left\{ \begin{array}{ll} {\varvec{\beta }}_i ={\varvec{\beta }}^0, &{} i=m+1, \dots , m + k^0_m; \\ {\varvec{\beta }}_i={\varvec{\beta }}^1 &{} i=m + k^0_m+1, \dots , m+T_m, \end{array} \right. \end{aligned}$$

where ${\varvec{\beta }}^0 \ne {\varvec{\beta }}^1$. The proposed test statistic, sensitive to the null hypothesis, is defined as

7 $$\begin{aligned} \mathcal {T}(m) = \sup _{1\leqslant k \leqslant T_m} \frac{\Vert \mathbf{{S}}(m,k) \Vert _\infty }{z(m,k,\gamma )}, \end{aligned}$$

for a standard supremum norm $\Vert \cdot \Vert _\infty$, a regularization function $z(m,k,\gamma ) \equiv m^{1/2}(1+k/m)(k/(k+m))^\gamma$ for some $\gamma \in [0, 1/2)$, and

8 $$\begin{aligned} \mathbf{{S}}(m,k) \equiv \textbf{J}^{-1/2}_m (\widehat{\varvec{\beta }}_m) \sum ^{m+k}_{i=m+1} \nabla \!f(\textbf{X}_i, \widehat{\varvec{\beta }}_m) g_\tau (\widehat{\varepsilon }_i), \end{aligned}$$

where $g_\tau (x) \equiv \rho '_\tau (x) = 2 \tau x \mathbb {I}_{\{x \ge 0\}}+2(1-\tau )x \mathbb {I}_{\{x<0\}}$ stands the first derivative of the expectile function $\rho _\tau (x)$ and $\widehat{\varepsilon }_i = Y_i - f(\varvec{X}_i, \widehat{\varvec{\beta }}_m)$ are so-called expectile residuals for $i = 1, \dots , m, m + 1, \dots , m + T_m$. Similarly, by $h_\tau (x) \equiv \rho ''_\tau (x) =2 \tau \mathbb {I}_{\{x \ge 0\}}+2(1-\tau ) \mathbb {I}_{\{x<0\}}$ we denote the second derivative of $\rho _\tau (x)$. In addition, $\nabla \!f(\textbf{X}_i, \widehat{\varvec{\beta }}_m)$ stands for a *p*-dimensional vector of the first partial derivatives $\frac{\partial }{\partial \varvec{\beta }}f(\varvec{X}_i, \widehat{\varvec{\beta }}_m)$ evaluated at the expectile estimate $\widehat{\varvec{\beta }}_m$, and

9 $$\begin{aligned} \textbf{J}_m (\widehat{\varvec{\beta }}_m)\equiv \frac{\mathbb {V}\text{ ar }\,[g_\tau (\varepsilon )]}{m} \sum ^m_{i=1} \nabla \! f(\textbf{X}_i,\widehat{\varvec{\beta }}_m) \nabla ^\top \!\! f(\textbf{X}_i,\widehat{\varvec{\beta }}_m), \end{aligned}$$

where $\textbf{J}^{-1/2}_m (\widehat{\varvec{\beta }}_m)$ in (8) denotes the inverse of the square root matrix (in a sense of the Cholesky factorization) of $\textbf{J}_m(\widehat{\varvec{\beta }}_m)$. A formal decision with respect to the null hypothesis in (5) is done by comparing the test statistic in (7) with the corresponding quantile of the limit distribution, which is a functional of a Wiener process (see Theorem 2). Details regarding the behaviour of the test statistic under the null hypothesis and the alternative hypothesis are derived in the next section.

### Remark 1

In practical applications, the theoretical quantity $\mathbb {V}\text{ ar }\,[g_\tau (\varepsilon )]$ in (9) is typically unknown. However, the corresponding finite sample counterpart $S_{g_{\hat{\tau }}}^2 = \frac{1}{(m - 1)} \sum _{i = 1}^m \big [g_{\hat{\tau }}(\widehat{\varepsilon }_i) \big ]^2$ can be used instead as a plug-in estimate, where $\hat{\tau } \in (0,1)$ (implicitly) solves $\frac{1}{m}\sum _{i = 1}^{m} g_{\tau }(\widehat{\varepsilon }_i) = 0$ (i.e., the empirical version of the theoretical assumption $\mathbb {E}[g_\tau (\varepsilon )] = 0$) and $\{\widehat{\varepsilon }_i\}_{i = 1}^m$ are the model-based residuals. The empirical estimates for the theoretical quantities $\mathbb {V}\text{ ar }\,[g_\tau (\varepsilon )]$, $\mathbb {E}[g_\tau (\varepsilon )]$, and $\tau \in (0,1)$ are all based on the historical data $\{(Y_i, \textbf{X}_i^\top )^\top ;~i = 1, \dots , m\}$.

## Theoretical results

Besides a *p*-dimensional vector $\nabla \! f({\textbf{x}},{\varvec{\beta }}) = \partial f({\textbf{x}}, {\varvec{\beta }})/ \partial {\varvec{\beta }}$, for any $\varvec{x} \in \varUpsilon$ and ${\varvec{\beta }}\in \Gamma$ let, analogously, $\nabla ^2 \! f({\textbf{x}},{\varvec{\beta }}) \equiv \partial ^2 f({\textbf{x}}, {\varvec{\beta }})/ \partial {\varvec{\beta }}^2$ be a $(p \times p)$-dimensional matrix of the second partial derivatives. In addition, let $\nabla ^2_j f({\textbf{x}},{\varvec{\beta }}) \equiv \big (\partial ^2 f({\textbf{x}}, {\varvec{\beta }})/(\partial \beta _l \partial \beta _j)\big )_{1 \leqslant l \leqslant p}$, which is again a *p*-vector for each $j \in \{1, \dots , p\}$. Finally, $\textbf{V}_m({\varvec{\beta }})$ stands for a $(p \times p)$-dimensional matrix being defined as $\textbf{V}_m({\varvec{\beta }}) \equiv m^{-1} \sum ^m_{i=1} \nabla \! f(\textbf{X}_i,{\varvec{\beta }}) \nabla ^\top \!\! f(\textbf{X}_i,{\varvec{\beta }})$ and for any two constants $a, b \in \mathbb {R}$ let $a \vee b= \max (a,b)$ and $a \wedge b= \min (a,b)$.

### Model assumptions

Considering the overall changepoint model in (1) and (2), the theoretical results formulated in this section rely on the set of assumptions stated below. For a better organization of the whole paper, the assumptions are split into five groups, (A)–(E).

ASSUMPTION **(A)**:

**(A1)**: The parameter space $\Gamma \subseteq \mathbb {R}^p$ is a compact set and the design space $\varUpsilon \subseteq \mathbb {R}^q$ is assumed to be bounded;
**(A2)**: For each $i \in \{1, \dots ,, m,m+1, m+T_m\}$, the partial derivatives $\nabla \! f(\textbf{X}_i,{\varvec{\beta }})$ and $\nabla ^2 \! f(\textbf{X}_i,{\varvec{\beta }})$ all exist and, moreover, $\nabla f(\textbf{X}_i,{\varvec{\beta }})$ is continuous on $\varUpsilon \times \Gamma$;
**(A3)**: For $q_m({\varvec{\beta }}) \equiv Card \{i \in \{1, \dots , m\}; f(\textbf{X}_i, {\varvec{\beta }}) \ne f(\textbf{X}_i, {\varvec{\beta }}^0)\}$ and every ${\varvec{\beta }}\in \Gamma$ such that ${\varvec{\beta }}\ne {\varvec{\beta }}^0$ it holds, that $0 < \lim _{m \rightarrow \infty } q_m/m \le 1$.

ASSUMPTION **(B)**: The density function of the random error terms $\{\varepsilon _i\}_{i = 1}^{m + T_m}$ (the generic error term $\varepsilon$ respectively) is continuous and strictly positive in zero.

ASSUMPTION **(C)**: There exists a positive definite matrix $\textbf{V}({\varvec{\beta }}^0)$ such that $\textbf{V}_m({\varvec{\beta }}^0)=m^{-1} \sum ^m_{i=1} \nabla \! f(\textbf{X}_i,{\varvec{\beta }}^0) \nabla ^\top \!\! f(\textbf{X}_i,{\varvec{\beta }}^0) \longrightarrow \textbf{V}({\varvec{\beta }}^0)$ for $m \rightarrow \infty$.

ASSUMPTION **(D)**: The model errors $\{\varepsilon _i\}_{i = 1}^{m + T_m}$ are independent and identically distributed (i.i.d.) with a continuous distribution, such that $\mathbb {E}[\varepsilon _i^4]< \infty$ and $\mathbb {E}[ g_\tau (\varepsilon _i)]=0$.

Assumptions (A), (B), and (C) are common conditions needed to show a strong consistency of the conditional expectile estimate $\widehat{\varvec{\beta }}_m$ defined in (4). Analogous conditions are used, for instance, by Choi et al (2003). Similarly, Assumption (D) is quite standard for the expectile models (e.g., Gu and Zou (2016), Kim and Lee (2016), or Ciuperca (2022)).

### Asymptotic behaviour of the expectile estimator

In order to study the asymptotic behaviour of the expectile estimator $\widehat{\varvec{\beta }}_m$ defined in (4) let us consider the *p*-square matrix

$$\begin{aligned} {\varvec{\Omega }}\equiv \mathbb {E}[h_\tau (\varepsilon )] \textbf{V}({\varvec{\beta }}^0). \end{aligned}$$

In addition to Assumption (A2), it is also required to impose slightly stricter assumptions on the matrix of the second partial derivatives $\nabla ^2 \! f({\textbf{x}},{\varvec{\beta }})$.

ASSUMPTION **(E)**: The elements of $\nabla ^2 \! f({\textbf{x}},{\varvec{\beta }})$ are all bounded for any ${\textbf{x}}\in \varUpsilon$ and for ${\varvec{\beta }}$ from a neighborhood of ${\varvec{\beta }}^0$ of radius of the order $m^{-1/2}$.

The assumption above is a common property which is—under Assumption (A1)—satisfied by any function *f* which is continuous on $\Upsilon \times \Gamma$. It is considered, for instance, for a sequential test in a nonlinear changepoint model in Ciuperca (2013) where an ordinary least squares (LS) estimation framework was used instead. For the expectile estimation framework proposed in this paper, the asymptotic behaviour of the estimator in (4) is formulated in the next proposition.

#### Proposition 1

Under Assumptions (A)–(E),

$$\begin{aligned} \widehat{\varvec{\beta }}_m={\varvec{\beta }}^0+{\varvec{\Omega }}^{-1} \frac{1}{m} \sum ^m_{i=1} \nabla \!f (\textbf{X}_i,{\varvec{\beta }}^0) g_\tau (\varepsilon _i)+o_\mathbb {P}(m^{-1/2}), \quad \text {as} m \rightarrow \infty . \end{aligned}$$

If the regression function *f* is linear in ${\varvec{\beta }}\in \Gamma$, then the asymptotic behaviour in the proposition reduces to a special case of Proposition 1 from Ciuperca (2022). Similarly, if the regression function *f* in (1) is nonlinear in ${\varvec{\beta }}\in \Gamma$, but the random error terms follow some normal distribution $N(0, \sigma ^2)$ with $\sigma ^2 < \infty$, the asymptotic behaviour in Proposition 1 gives the results of Theorem 2.1 in Seber and Wild (2003).

### Test statistic under $H_0$ and $H_1$

The asymptotic behaviour of the test statistic defined in (7) is investigated in this section under both—the null hypothesis in (5) and the alternative hypothesis in (6). Note that that the vectors of parameters ${\varvec{\beta }}^0, {\varvec{\beta }}^1\in \Gamma$, where ${\varvec{\beta }}^0 \ne {\varvec{\beta }}^1$, are both unknown. Let $\textbf{J}_m ({\varvec{\beta }})\equiv \mathbb {V}\text{ ar }\,[g_\tau (\varepsilon )]\textbf{V}_m({\varvec{\beta }})$, for ${\varvec{\beta }}\in \Gamma$, be a $p \times p$ matrix—a theoretical (deterministic) version of its empirical counterpart—the $p \times p$ matrix $\textbf{J}_m (\widehat{\varvec{\beta }}_m)$ defined in (9). Considering the size $m \in \mathbb {N}$ for the *historical data* and the size $T_m \in \mathbb {N}$ for the *online data* there are two specific possibilities which should be considered separately.

- if $\lim _{m \rightarrow \infty } T_m/m=\infty$ for either $T_m=\infty$ or $T_m < \infty$, then such a scenario is called an *open-end procedure*;
- if $\lim _{m \rightarrow \infty } T_m/m=T$ for $T_m < \infty$ where $T \in (0, \infty )$, then such a scenario is called a *closed-end procedure*.

By a common convention, it is usually assumed that for the open-end procedures it holds that $T=\infty$.

#### Theorem 2

Let Assumptions (A)–(E) be satisfied. Then, under $H_0$,

$$\begin{aligned} \mathcal {T}(m) \equiv \sup _{1\leqslant k \leqslant T_m} \frac{\Vert \mathbf{{S}}(m,k) \Vert _\infty }{z(m,k,\gamma )} \overset{\mathcal{L}}{\underset{m \rightarrow \infty }{\longrightarrow }} \sup _{0< t < L(T) } \frac{\Vert {\textbf {W}}_p(t)\Vert _\infty }{t^\gamma }, \end{aligned}$$

where $\{{\textbf {W}}_p(t); \; t \in (0,\infty )\}$ is a *p*-dimensional Wiener process where $L(T)=1$ for the open-end procedure and $L(T)=T/(1+T)$ for the closed-end procedure.

The test statistic in Theorem 2 is based on the expectile estimator $\widehat{\varvec{\beta }}_m$ of the true parameter vector ${\varvec{\beta }}^0 \in \Gamma$ calculated from the *historical data*. However, the limit process is the same as for the expectile estimator in the linear model considered in Ciuperca (2022), or the quantile estimator proposed in Zhou et al (2015). On the other hand, the test statistic is different from that proposed by Ciuperca (2013) or Horváth et al (2004) where the authors rather considered the CUSUM type statistic based on the least squares residuals of the linear model or the nonlinear model respectively.

In addition, the asymptotic behaviour of the test statistic under the null hypothesis in Theorem 2 does not depend on the underlying form of the nonlinear regression function *f* nor the true value ${\varvec{\beta }}^0$ as was the case for the test statistic applied for the parametric nonlinear model proposed in Ciuperca (2013). Therefore, the test statistic in Theorem 2 generally less restrictive, it is easier to use, and more straightforward to apply also for the least squares estimation (i.e., when $\tau =1/2$).

For the behaviour of the test statistic under the alternative hypothesis, more caution is needed. The model in (1) changes after the *historical data* and this change must be identifiable. Consequently, some reasonable assumptions are needed for the difference between the true parameter vectors ${\varvec{\beta }}^0$ and ${\varvec{\beta }}^1$ and, also, the underlying regression function *f*. Specific details are formulated in the next theorem.

#### Theorem 3

Let Assumptions (A)–(E) be satisfied and let $m^{1/2}\Vert {\varvec{\beta }}^0 - {\varvec{\beta }}^1\Vert _2 \rightarrow \infty$ as $m \rightarrow \infty$. If there exists $C>0$ such that

$$\begin{aligned} \frac{1}{m^s} \left\| \sum ^{m+\widetilde{k}_m}_{i=m+k^0_m+1} c_i \nabla \!f(\textbf{X}_i, {\varvec{\beta }}^0) \big [ f(\textbf{X}_i, {\varvec{\beta }}^1) - f(\textbf{X}_i, {\varvec{\beta }}^0)\big ]\right\| _\infty>C>0 \end{aligned}$$

for some constants $|c_i | \in [2(\tau \wedge (1-\tau )), 2(\tau \vee (1-\tau ))]$, then

$$\begin{aligned} \sup _{1\leqslant k \leqslant T_m} \frac{\Vert \mathbf{{S}}(m,k) \Vert _\infty }{z(m,k,\gamma )} \overset{\mathbb {P}}{\underset{m \rightarrow \infty }{\longrightarrow }} \infty . \end{aligned}$$

Considering the assertions of both theorems together, the statistical test based on the proposed test statistics in (7) is proved to be consistent. The decision rule can be defined directly by considering the corresponding quantiles of the limit process from Theorem 2.

#### Example 1

For a simple linear function $f(x, {\varvec{\beta }})=\beta _0+\beta _1 x$, the unknown vector parameters ${\varvec{\beta }}^0=(\beta ^0_1, \beta ^0_2)^\top \in \Gamma$ and ${\varvec{\beta }}^1=(\beta ^1_1, \beta ^1_2)^\top \in \Gamma$, and $x \in \Upsilon \subseteq \mathbb {R}$ one just need that $\Gamma \subseteq \mathbb {R}^2$ is a compact set, $\Upsilon$ is bounded, and $(\beta ^1_1-\beta ^0_1) (\beta ^1_2-\beta ^0_2) \ne 0$ for the assumptions in (A1)–(A3) to hold. Assumption (B) is typically valid for common (continuous) error distributions. Assumptions (C) and (E) are satisfied trivially by the linearity of *f*. Finally, Assumption (D) can not be usually verified in a straightforward way but a sample estimate for $\tau \in (0,1)$ can be used such that the empirical counterpart of the equation $\mathbb {E}[g_\tau (\varepsilon _i)] = 0$ is satisfied.

#### Example 2

For a nonlinear function $f(x, {\varvec{\beta }}) = \exp \{ - \beta _1 e^{- \beta _2 x}\}$ (the Gompertz curve for $x \in \Upsilon$) with ${\varvec{\beta }}^0=(\beta ^0_1, \beta ^0_2)^\top \in \Gamma$ and ${\varvec{\beta }}^1=(\beta ^1_1, \beta ^1_2)^\top \in \Gamma$ for $\Gamma = (0, \infty )\times (0, \infty )$ and some bounded $\Upsilon \subset \mathbb {R}$ it is easy to see that $\nabla \!f(x, {\varvec{\beta }}) = (- f(x, {\varvec{\beta }}) e^{-\beta _2 x}, f(x, {\varvec{\beta }}) \beta _1 x e^{-\beta _2 x})^\top$ is continuous on $\Upsilon \times \Gamma$ and $\nabla ^2 f(x, {\varvec{\beta }})$ exists. Thus, for (A1)–(A3) to hold, one just needs that $(\beta ^1_1-\beta ^0_1) (\beta ^1_2-\beta ^0_2) \ne 0$. Assumption (C) can be shown in a straightforward way and the remaining assumptions are analogous to Example 1.

On the basis of the results obtained above one can define a stopping time—i.e., the first observation for which the null hypothesis in (5) is rejected in favor of the alternative hypothesis—considering the significance level $\alpha \in (0,1)$. The corresponding changepoint estimate is defined as

$$\begin{aligned} \widehat{k}_m \equiv \left\{ \begin{array}{l} \inf \Big \{ k \ge 1; \; \; \sup _{1\leqslant k \leqslant T_m} \frac{\Vert \mathbf{{S}}(m,k) \Vert _\infty }{z(m,k,\gamma )} > c_{\alpha }(\gamma ) \Big \}; \\ \\ \infty , \qquad \qquad \text {if } \sup _{1\leqslant k \leqslant T_m} \frac{\Vert \mathbf{{S}}(m,k) \Vert _\infty }{z(m,k,\gamma )} \le c_{\alpha }(\gamma ) \text { for all } k=1, \dots , T_m, \end{array} \right. \end{aligned}$$

where $c_{\alpha }(\gamma )$ is $(1- \alpha )$-quantile of the distribution of $\sup _{0< t < L(T) } {\Vert {\textbf {W}}_p(t)\Vert _\infty }/{t^\gamma }$. Note, that $\widehat{k}_m$ is the corresponding index referring to the online data only (i.e., $\widehat{k}_m \in \{1, \dots , T_m\}$). Thus, from the overall point of view, the underlying model changes after $m + \widehat{k}_m$ observations. It holds that $\lim _{m \rightarrow \infty } \mathbb {P}[\widehat{k}_m < \infty \mid H_0 \text { true} ]=\alpha$ and, similarly, $\lim _{m \rightarrow \infty } \mathbb {P}[\widehat{k}_m < \infty \mid H_1 \text { true} ]=1$. Hence, the proposed test is consistent.

## Empirical study

Finite sample properties of the proposed real-time changepoint detection method based on the expectile estimator defined in (4) are closely investigated in this section. Firstly, the empirical level of the test is assessed under various settings and the empirical power of the test is investigated for various changepoint scenarios. In the second part, the proposed methodology is also applied to analyze the Covid-19 prevalence data from Prague, Czech Republic, in order to link some authorities’ decisions to the real-time pandemic situation.

### Simulation experiment

**Fig. 1:**
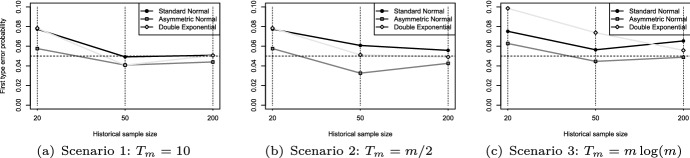
The asymptotic behavior of the empirical Type I error probabilities for three different values of $m \in \{20, 50, 200\}$, three error distributions (standard normal, asymmetric normal with unit mean and variance, and double exponential with zero mean and unit variance) and three different choices of $T_m$ in order to mimic the closed-end and open-end scenarios

The main concept of the simulation study is analogous to that presented in Choi et al (2003). However, instead of a simple exponential function used for the underlying regression, a more complex Gompertz curve of the form

$$\begin{aligned} f(x, \varvec{\beta }) = \exp \{- \beta _1 e^{-\beta _2 x}\} \end{aligned}$$

is employed, where $\varvec{\beta }^0 = (\beta _1, \beta _2)^\top \equiv (10, 5)^\top$ and $x \in (0,1)$. The reason is that the function used in Choi et al (2003) becomes very insensitive to any parameter change for large $x_t = t$ (even for $t \ge 10$). A simple *iterative grid search algorithm* is implemented to solve (4) and the changepoint test is performed in terms of Theorem 2. For the length of the historical period there are three different options considered ($m \in \{20, 50, 200\}$). Analogously as in Choi et al (2003), three error distributions are used: a symmetric standard normal distribution (with $\tau = 0.5$), asymmetric normal distribution with the mean and variance being equal to one ($\hat{\tau } = 0.0719$), and, finally, a heavy-tailed (symmetric) Laplace distribution with the zero mean and unit variance (again, $\tau = 0.5$ due to the symmetric property). In order to mimic both situations—the closed-end scenario and the open-end scenario—there are again tree options considered for $T_m \in \{10, m/2, m \log m \}$. The empirical results under the null hypothesis (of no change in the model) are summarized in Table 1 and in Fig. 1. Different values for the regularization parameter $\gamma \in [0, 1/2)$ were considered as well but no substantial differences were found, therefore, all reported results are for $\gamma = 0.1$ only.

**Table 1:** Simulation results under the null hypothesis (with the theoretical value of $\tau = 0.5$ for the symmetric distributions and the empirical estimate $\hat{\tau } = 0.0719$ in terms of Remark 1 for the asymmetric distribution)

Distribution	*m*	${\widehat{\beta }}_1 \mid$ Std.Er.	${\widehat{\beta }}_2 \mid$ Std.Er.	Scenario 1	Scenario 2	Scenario 3
		$\beta _1 = 10.00$	$\beta _2 = 5.00$	$T_m = 10$	$T_m = m/2$	$T_m = m \log (m)$
*N*(0, 1)	20	10.52 *(2.832)*	5.32 *(1.825)*	7.74%	7.74%	7.52%
$\tau = 0.5000$	50	10.32 *(2.857)*	5.20 *(1.382)*	4.92%	6.08%	5.64%
	200	10.24 *(2.766)*	5.03 *(0.808)*	5.08%	5.58%	6.54%
*N*(1, 1)	20	10.18 *(2.889)*	5.61 *(2.020)*	5.76%	5.76%	6.28%
$\hat{\tau } = 0.0719$	50	10.27 *(2.869)*	5.39 *(1.638)*	4.08%	3.26%	4.46%
	200	10.30 *(2.833)*	5.08 *(0.977)*	4.40%	4.26%	4.88%
*L*(0, 1)	20	10.50 *(2.842)*	5.32 *(1.797)*	7.84%	7.84%	9.86%
$\tau = 0.5000$	50	10.37 *(2.850)*	5.18 *(1.374)*	4.08%	5.12%	7.38%
	200	10.29 *(2.769)*	5.02 *(0.799)*	5.06%	4.90%	5.58%

The parameter estimates are reported with the corresponding standard errors (in parentheses) over 5000 Monte Carlo simulations. Relative proportions of false rejections are given for three different scenarios for $T_m$ reflecting the open-end and closed-end procedures. The nominal level of all the tests is always set to be $\alpha = 0.05$

The empirical level of the test seems to properly keep the nominal level of $\alpha = 0.05$ for all considered scenarios. The results are slightly conservative for the symmetric distributions (the normal distribution *N*(0, 1) and the double exponential distribution *L*(0, 1)). On the other hand, a slightly underestimated nominal level is observed for the asymmetric error distribution (the normal distribution *N*(1, 1)) but the actual differences are rather negligible. The corresponding expectile estimates of the unknown (true) parameters $\beta _1 = 10$ and $\beta _2 = 5$ seem both to be consistent for all considered scenarios and no inconsistences are observed in Table 1.

**Table 2:** Empirical powers of the proposed real-time changepoint test based on 5000 Monte Carlo simulations given for various simulation settings

Dist.	*m*	${k}_m^{0}$	Scenario 1		Scenario 2		Scenario 3	
			$T_m = 10$		$T_m = m/2$		$T_m = m \log (m)$	
*N*(0, 1)	20	$k_m^{(1)}$	2.79%	[0.77 $\mid$ 0.78]	2.79%	[0.77 $\mid$ 0.78]	2.44%	[0.72 $\mid$ 0.71]
$\tau = 0.5000$		$k_m^{(2)}$	12.80%	[0.31 $\mid$ 0.22]	12.80%	[0.31 $\mid$ 0.22]	20.40%	[0.19 $\mid$ 0.12]
	50	$k_m^{(1)}$	4.10%	[0.73 $\mid$ 0.76]	4.10%	[0.76 $\mid$ 0.78]	5.20%	[0.93 $\mid$ 0.93]
		$k_m^{(2)}$	13.30%	[0.33 $\mid$ 0.11]	14.60%	[0.44 $\mid$ 0.46]	35.30%	[0.32 $\mid$ 0.25]
	200	$k_m^{(1)}$	6.52%	[0.87 $\mid$ 0.89]	8.99%	[0.80 $\mid$ 0.82]	21.76%	[0.83 $\mid$ 0.85]
		$k_m^{(2)}$	16.70%	[0.46 $\mid$ 0.44]	47.60%	[0.46 $\mid$ 0.49]	96.40%	[0.15 $\mid$ 0.10]
*N*(1, 1)	20	$k_m^{(1)}$	2.51%	[0.71 $\mid$ 0.67]	2.51%	[0.71 $\mid$ 0.67]	2.42%	[0.62 $\mid$ 0.55]
$\hat{\tau } = 0.0719$		$k_m^{(2)}$	11.20%	[0.37 $\mid$ 0.39]	11.20%	[0.37 $\mid$ 0.39]	23.30%	[0.29 $\mid$ 0.22]
	50	$k_m^{(1)}$	4.20%	[0.83 $\mid$ 0.83]	4.30%	[0.83 $\mid$ 0.83]	5.00%	[0.85 $\mid$ 0.89]
		$k_m^{(2)}$	12.20%	[0.43 $\mid$ 0.46]	13.30%	[0.49 $\mid$ 0.33]	32.30%	[0.48 $\mid$ 0.58]
	200	$k_m^{(1)}$	5.52%	[0.82 $\mid$ 0.84]	7.20%	[0.55 $\mid$ 0.55]	21.71%	[0.83 $\mid$ 0.85]
		$k_m^{(2)}$	14.21%	[0.42 $\mid$ 0.44]	38.00%	[0.48 $\mid$ 0.49]	93.80%	[0.18 $\mid$ 0.15]
*L*(0, 1)	20	$k_m^{(1)}$	2.64%	[0.77 $\mid$ 0.78]	2.64%	[0.77 $\mid$ 0.78]	2.29%	[0.74 $\mid$ 0.78]
$\tau = 0.5000$		$k_m^{(2)}$	11.50%	[0.36 $\mid$ 0.22]	11.50%	[0.36 $\mid$ 0.22]	21.20%	[0.24 $\mid$ 0.16]
	50	$k_m^{(1)}$	3.95%	[0.75 $\mid$ 0.78]	4.10%	[0.78 $\mid$ 0.79]	4.99%	[0.81 $\mid$ 0.86]
		$k_m^{(2)}$	12.90%	[0.43 $\mid$ 0.44]	13.20%	[0.48 $\mid$ 0.50]	37.70%	[0.30 $\mid$ 0.21]
	200	$k_m^{(1)}$	6.70%	[0.75 $\mid$ 0.67]	6.93%	[0.80 $\mid$ 0.79]	24.04%	[0.81 $\mid$ 0.83]
		$k_m^{(2)}$	15.60%	[0.40 $\mid$ 0.47]	37.90%	[0.46 $\mid$ 0.47]	96.40%	[0.18 $\mid$ 0.12]

The change in $\beta _2$ occurs either in the first half of the online data for $k_m^{(1)} = \lfloor T_m / 2\rfloor + 1$, or the change occurs at the very beginning of the online data, thus $k_m^{(2)} = 1$. The empirical powers for different $T_m$, different error distributions, and three sizes of the historical data are given in terms of the relative proportions (using $\tau = 0.5$ for the symmetric distributions and $\hat{\tau } = 0.0719$ for the asymmetric distribution). Two changepoint indicators are also used in the brackets: the average changepoint location index and the median changepoint location index. Values close to zero stand for an early changepoint detection (zero standing for the detection at the first available observation) and values close to one mean late changepoint discoveries (one standing for the detection at the last available observation)

On the other hand, the situation under the alternative hypothesis becomes slightly more comprehensive as there might be many different changepoint scenarios to possibly consider and take into account. For brevity purposes, there are only the results for one representative situation provided in this manuscript, but,any other situations were considered and compared with rather analogous results among all.

In particular, the following simulation scenarios under the alternative hypothesis were considered:

- A change occurs either in $\beta _1$, or in $\beta _2$, or in both elements of $\varvec{\beta } = (\beta _1, \beta _2)^\top$ simultaneously;
- A change occurs immediately after the historical data or the changepoint occurs after the first half of the online data;
- The magnitude of the change is relatively small compared to the true parameter values ($20\%$ change with respect to the true value) or the change is relatively large (the parameter(s) after the changepoint is(are) doubled);
- Finally, if the changepoint occurs in both elements of $\varvec{\beta } = (\beta _1, \beta _2)^\top$, the corresponding effects of the changes may act against each other—thus, the resulting regression function after the change is very similar to the regression function before the change—or, alternatively, the effects of the changes aim at the same direction—thus, the regression function after the change is quite different from the underlying regression function before the change and there is also more power in the data to reveal such change.

All these situations have, of course, an important impact on the simulation results and, in particular, the performance of the proposed test in terms of its empirical power. For illustration purpose, one particular scheme (with the changepoint in $\beta _2$ only and the change magnitude being equal to the true value of $\beta _2$) is reported in Table 2. It is obvious from the table, that the performance of the proposed test (in terms of the empirical power) mostly depends on the true changepoint location and the length of the *online data* but in all considered situations the proposed test seems to be consistent.

**Fig. 2:**
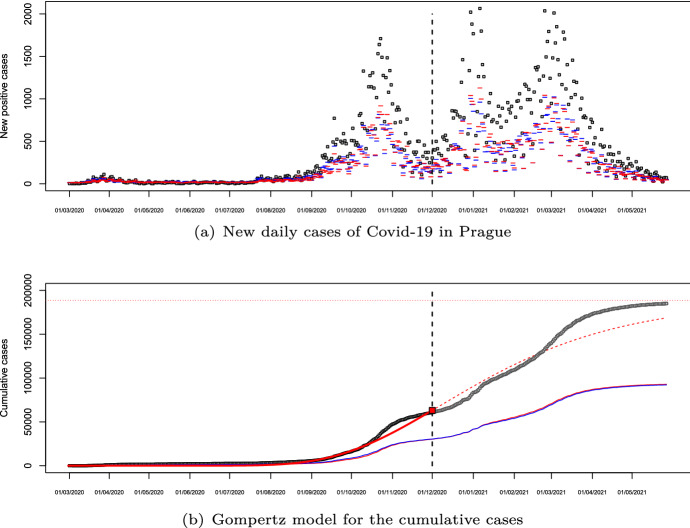
Covid-19 positive cases in Prague, Czech Republic. The overall daily increments in the upper panel and the cumulative counts in the lower panel are—just for better illustration—provided also separately for males (blue) and females (red). The vertical lines represent the date when the strict pandemic restrictions being in effect before Christmas 2020 were relaxed. The Gompertz population model in (10) is fitted on the *historical data*—the data before the restrictions release in December 1, 2020. The projection of the model is provided for the future in dashed red. The estimated saturation of $\widehat{K} = 189~616$ is visualized in doted red

Note that for the situations where the changepoint occurs in the first half of the *online data* (the rows denoted as $k_m^{(1)}$ in Table 2), there are some false rejections (roughly $5\%$) of the observed rejections in the first half of the online data before the actual change appears. Such false rejection are not considered in Table 2 and only the rejections after the first half of the *online data* are reported. This is also reflected by the fact that the average and median *changepoint location indicators* in the brackets are always greater than 0.50—which stands for the half of the *online data* sequence.

The average changepoint location indicator of, let us say, 0.25 indicates that the changepoint was estimated (when averaged over all simulations) after the first quarter of the *online data*. If the median location indicator (the second value in the brackets) is higher than the average, then the majority of the changepoint recoveries occurred after the first quarter, but there were also some relatively rear although very early recoveries (including also the very first online observation). On the other hand, for the median location indicator being smaller than the average indicator, the majority of the changepoint recoveries occurred before the first quarter, but there were also some very late recoveries (including the very last observations).

### Covid-19 prevalence

Relatively recently, the world society was very much effected by the Covid-19 pandemic, therefore, we tried to apply the proposed estimation and changepoint detection method for a nonlinear parametric population risk model—a three parameter Gompertz curve—to model the cumulative counts of the Covid-19 positive cases in Prague, the capital of the Czech Republic, over the period from the first positive case appearance (March 1, 2020) until the end of May 2021. The data, provided for academic purposes by the Institute of Health Information and Statistics of the Czech Republic are assumed to follow a typical nonlinear (growth) model in (1), where

10 $$\begin{aligned} f(\varvec{X}_i, {\varvec{\beta }}) = K \exp \Big \{ -\beta _1 e^{- \beta _2 x_i} \Big \} \end{aligned}$$

for the unknown parameter vector $\varvec{\beta } = (\beta _1, \beta _2, K)^\top \in \mathbb {R}_{+}^{3}$. The univariate explanatory variables $\varvec{X}_{i} \equiv x_i$ stand for the current day and the dependent random variables $Y_i$ in (1) reflect the cumulative Covid-19 positive cases at the given day. A similar population growth model—a five parameter logistic curve—was recently applied in Chen et al (2020) to predict the overall number of positive Covid-19 cases in the US. The resulting model, however, turned out to heavily underestimate the true number of positive cases, which could be also caused by the underlying distributional symmetry assumption.

**Table 3:** Parameter estimates for the underlying Gompertz model in (10) for three different data scenarios: firstly, historical data until restrictions release are considered; Second, the proposed online testing procedure is applied until the null hypothesis is rejected and the model is retrained. Finally, the all available data are used to estimate the overall model

Estimation method and data	${\beta }_1$	${\beta }_2$	*K*	Objective function
*Historical data (until 01/12/2020)*				
Symmetric least squares ($\tau = 0.50$)	36.04	0.0129	187 811	$3.00 \times 10^6$
Expectile method ($\tau = 0.11$)	37.97	0.0129	188 576	$0.78 \times 10^6$
*Re-estimation after the change detection (03/12/2020)*				
Symmetric least squares ($\tau = 0.50$)	35.38	0.0130	180 174	$3.27 \times 10^6$
Expectile method ($\tau = 0.19$)	36.04	0.0129	179 718	$1.92 \times 10^6$
*All available data (until 26/05/2021)*				
Symmetric least squares ($\tau = 0.50$)	20.17	0.0096	256 970	$10.9 \times 10^6$
Expectile method ($\tau = 0.26$)	20.18	0.0095	255 032	$8.53 \times 10^6$

**Fig. 3:**
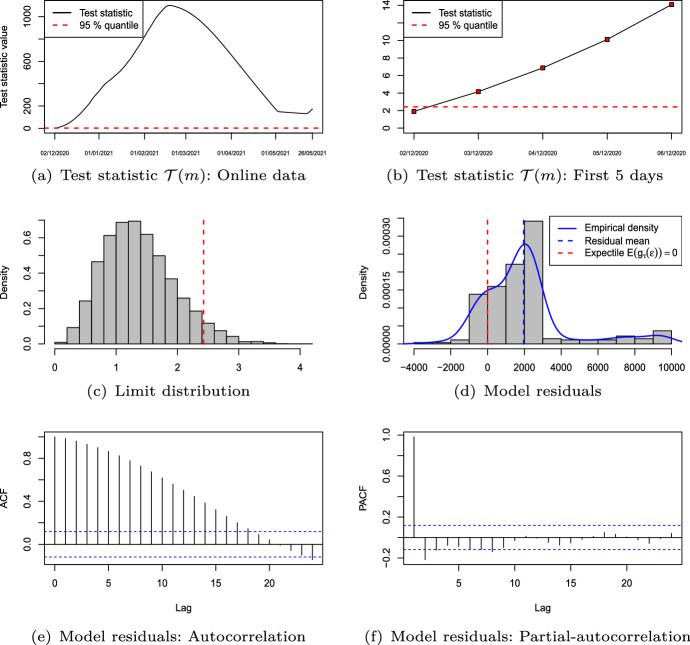
The test statistic profile for the *online data* in panel (**a**) and the first five days only for a more detailed insight in panel (**b**); The limit distribution from Theorem 2 with the corresponding $95\%$ sample quantile $c_{0.95}(\gamma ) = 2.4260$, for $\gamma = 0.1$, in panel (**c**); The model residuals from (10) with the corresponding density estimate, the empirical mean, and the empirical expectile for $\widehat{\tau } = 0.11$, such that the empirical conterpart of $E[g_{\tau }(\varepsilon )]$ equals to zero—all in panel (**d**); Finally, the residual autocorrelation and partial-autocorrelation plots in panels (**e**) and (**f**) respectively

In our approach, instead of trying to predict the overall positive cases, we pursue a slightly different goal: Firstly, the data are split into two parts—the *historical data* from the very first Covid-19 positive case in Prague until December 1, 2020 (when a rather populistic and quite much criticized government decision waved off some of the strict pandemic restrictions before Christmas) and the *online data*—arriving after December 1, 2020. Second, the proposed changepoint test is adopted to test whether the model before the government decision and the model after the government decision is the same, or not. Finally, the model can be also used to get some predictions of the overall Covid-19 positive cases over the overall follow-up period.

The data—daily positive cases—are visualized in Fig. 2a. The corresponding cumulative counts are given in the panel below—Fig. 2b. The Gompertz model from (10) is fitted on the *historical data* thus, the period from March 1, 2020 until December 1, 2020. The estimated parameters are provided in Table 3. The estimated number of the overall Covid-19 positive cases is $\widehat{K} = 188~576$, while the true number of all positive cases reported until May 26, 2021, is 184 959.

The proposed changepoint detection test based on (7) is performed to verify the stability of the model trained on the *historical data*, for $m = 275$, while new *online data* are arriving in a step-by-step manner (for $T_m = 176$). The values for the test statistic in (7) at each step of the online testing regime are plotted in Fig. 3a. The null hypothesis of no changepoint in the vector parameter $\varvec{\beta } = (\beta _1, \beta _2, K)^\top$ is rejected relatively fast—just two days after the government reduced the restrictions—the corresponding test statistic is $\mathcal {T}(m) = 4.1618$ for $m = 275$ and the corresponding $95\%$ quantile of the limit distribution from Theorem 2 is $c_{0.95}(\gamma ) = 2.4260$ for $\gamma = 0.1$. This may suggest that the actual change in the model occurred already before the online data—which can be also seen in Fig. 2—either from the first peak and the consecutive drop-off in panel (a) or some evident underestimation at the end of the historical data in panel (b). The estimated parameters for the retrained model after the changepoint detection are, for comparison, also reported in Table 3. Alternatively, one could also consider another set of the historical data (and maybe slightly more representative)—from the very first case until the first culmination (i.e., the beginning of November 2020, thus $m = 245$) and to test whether the model changes significantly after the peak as the daily Covid-19 cases start to decrease. The estimated parameters are very similar ($\widehat{\beta _1} = 88.15$, $\widehat{\beta }_2 = 0.0166$, and $\widehat{K} = 197 264$) but it takes 8 days for the proposed test statistic to detect a significant change in the model. Nevertheless, despite some obvious correlation among the model-based residuals (Fig. 3d and e) the estimated model seems to be relatively stable and the proposed changepoint detection test performs very well.

## Conclusions

In this paper, we proposed the *online procedure* for testing stability of a nonlinear parametric regression model while taking into account the conditional expectile estimation framework. There are three main pivots behind the proposed methodology: Firstly, the nonlinear parametric form of the unknown regression function improves the overall flexibility of the model while the dependence on the unknown parameters still preserves a relatively simple and straightforward interpretation of the overall regression function estimate. Second, the expectile estimation method allows for some additional robustness especially with respect to asymmetric distributions. The estimation algorithm depends on the “asymmetry index” $\tau \in (0, 1)$, which is usually unknown, but it can be either anticipated from the data generating mechanism or some plug-in estimate can be used instead. Third, the online regime for the changepoint detection makes the proposed method instantly applicable, which may turn out to be convenient in situations when real-time decisions and model adaptations are required. Finally, given the underlying regression function, the whole minimization problem formulated in (4) does not have to be convex—therefore, we proposed a widely applicable general iterative grid search algorithm which can be effectively used in practical applications.

The proposed methodological framework enriches the class of *online procedures* for changepoint detections. To our best knowledge, the specific model setup considered in this paper has not been studied in the literature yet. The empirical performance is illustrated through an extensive simulation study. A practical applicability of the whole methodological framework is illustrated on a real data example concerning some of the most recent challenges related to online decision making—especially essential decisions related to the Covid-19 pandemics made by local and global authorities.

## Appendix A Proofs and technical lemma

### Proof of Proposition 1

The expectile estimator $\widehat{\varvec{\beta }}_m$ from (4) is the solution of the system of equations

$$\begin{aligned} \frac{\partial }{ \partial {\varvec{\beta }}} \sum ^m_{i=1} \rho _\tau \big (Y_i-f(\textbf{X}_i, {\varvec{\beta }})\big ) ={\textbf {0}}_p, \end{aligned}$$

where ${\textbf {0}}_p$ stands for a zero vector of dimension *p*. Then, with a probability equal to 1, it holds that

A1 $$\begin{aligned} \sum ^m_{i=1} \nabla \!f (\textbf{X}_i,\widehat{\varvec{\beta }}_m) g_\tau (\varepsilon _i+f(\textbf{X}_i,{\varvec{\beta }}^0)- f(\textbf{X}_i,\widehat{\varvec{\beta }}_m)) ={\textbf {0}}_p. \end{aligned}$$

Since under Assumptions (A), (B), (C) we have $\widehat{\varvec{\beta }}_m \overset{{a.s.}}{\underset{m \rightarrow \infty }{\longrightarrow }} {\varvec{\beta }}^0$ (see Theorem 2.2 of Choi et al (2003)) and since for $t \rightarrow 0$ we have $g_\tau (\varepsilon -t)=g_\tau (\varepsilon ) - h_\tau (\varepsilon ) t+o_\mathbb {P}(t)$, then using Assumptions (A1) and (A2), we obtain

$$\begin{aligned} g_\tau \Big (\varepsilon _i+f(\textbf{X}_i,{\varvec{\beta }}^0)- f(\textbf{X}_i,\widehat{\varvec{\beta }}_m)\Big )= & {} g_\tau (\varepsilon _i) - h_\tau (\varepsilon _i) \big (f(\textbf{X}_i,\widehat{\varvec{\beta }}_m) - f(\textbf{X}_i,{\varvec{\beta }}^0)\big )\\{} & {} + o_\mathbb {P}\big (f(\textbf{X}_i,\widehat{\varvec{\beta }}_m) - f(\textbf{X}_i,{\varvec{\beta }}^0) \big ). \end{aligned}$$

Substituting into the relation in (A1) we obtain

$$\begin{aligned} {\textbf {0}}_p{} & {} =\sum ^m_{i=1} \nabla \!f(\textbf{X}_i,\widehat{\varvec{\beta }}_m) \bigg [g_\tau (\varepsilon _i) - h_\tau (\varepsilon _i) \big (f(\textbf{X}_i,\widehat{\varvec{\beta }}_m) - f(\textbf{X}_i,{\varvec{\beta }}^0)\big ) \\{} & {} \quad +o_\mathbb {P}\big (f(\textbf{X}_i,\widehat{\varvec{\beta }}_m) - f(\textbf{X}_i,{\varvec{\beta }}^0)\big ) \bigg ] \end{aligned}$$

and, since $\widehat{\varvec{\beta }}_m \overset{{a.s.}}{\underset{m \rightarrow \infty }{\longrightarrow }} {\varvec{\beta }}^0$, using also Assumption (A2) and the Taylor expansion, we have, for any $j=1, \dots , p$, that

$$\begin{aligned} 0= & {} \sum ^m_{i=1} \bigg [\frac{\partial f}{\partial \beta _j} (\textbf{X}_i, {\varvec{\beta }}^0) + \nabla ^2_j f^\top (\textbf{X}_i, {\varvec{\beta }}^0+\theta _{ji}(\widehat{\varvec{\beta }}_m- {\varvec{\beta }}^0)) (\widehat{\varvec{\beta }}_m- {\varvec{\beta }}^0) \bigg ] \\{} & {} \times \bigg [ g_\tau (\varepsilon _i) - h_\tau (\varepsilon _i) \big (f(\textbf{X}_i,\widehat{\varvec{\beta }}_m) - f(\textbf{X}_i,{\varvec{\beta }}^0)\big ) +o_\mathbb {P}\big (f(\textbf{X}_i,\widehat{\varvec{\beta }}_m) - f(\textbf{X}_i,{\varvec{\beta }}^0)\big )\bigg ], \end{aligned}$$

with $\nabla ^2_j f$ the column *p*-vector $\big (\partial ^2 f / (\partial \beta _j \partial \beta _k)\big )_{1 \leqslant k \leqslant p}$ of derivatives of order 2 and $\theta _{ji}$ a random variable such that $0 \le \theta _{ji} \le 1$ with probability 1. Hence, using Assumptions (A1), (A2), (C), and (E), together with the Cauchy–Schwarz inequality and the Taylor expansion, we obtain

A2 $$\begin{aligned} {\textbf {0}}_p&=\frac{1}{m} \sum ^m_{i=1}\nabla \!f(\textbf{X}_i,{\varvec{\beta }}^0)g_\tau (\varepsilon _i) - \frac{1}{m} \sum ^m_{i=1} h_\tau (\varepsilon _i)\nabla \!f(\textbf{X}_i,{\varvec{\beta }}^0) \nabla \!f^\top (\textbf{X}_i,{\varvec{\beta }}^0)(\widehat{\varvec{\beta }}_m - {\varvec{\beta }}^0) \nonumber \\&\quad +o_\mathbb {P}(\widehat{\varvec{\beta }}_m - {\varvec{\beta }}^0). \end{aligned}$$

By Assymption (C) we can define a *p*-square invertible matrix

$$\begin{aligned} {\varvec{\Omega }}_m \equiv m^{-1} \sum ^m_{i=1} h_\tau (\varepsilon _i)\nabla \!f(\textbf{X}_i,{\varvec{\beta }}^0) \nabla \!f^\top (\textbf{X}_i,{\varvec{\beta }}^0) \end{aligned}$$

and using the relation in (A2) we obtain

A3 $$\begin{aligned} \widehat{\varvec{\beta }}_m - {\varvec{\beta }}^0 ={\varvec{\Omega }}_m^{-1} \frac{1}{m} \sum ^m_{i=1}\nabla \!f(\textbf{X}_i,{\varvec{\beta }}^0) g_\tau (\varepsilon _i)\big (1+o_\mathbb {P}(1)\big ), \end{aligned}$$

or, again, using Assumptions (C) and (D) to get

$$\begin{aligned} \widehat{\varvec{\beta }}_m = {\varvec{\beta }}^0 +{\varvec{\Omega }}^{-1} \frac{1}{m} \sum ^m_{i=1}\nabla \!f(\textbf{X}_i,{\varvec{\beta }}^0) g_\tau (\varepsilon _i)\big (1+o_\mathbb {P}(1)\big ), \end{aligned}$$

which proves the given proposition. $\square$

In order to show the asymptotic behavior of the test statistic under the null hypothesis in (5) and the alternative hypothesis in (6) let us define a stochastic process

A4 $$\begin{aligned} {\textbf{r}}_{m,k}(\mathbf {{u}}) \equiv \sum ^{m+k}_{i=m+1} {\textbf{R}}_i(\mathbf {{u}}), \end{aligned}$$

for $k =1, \ldots , T_m$, where $\mathbf {{u}}\in \mathbb {R}^p$, such that $\Vert \mathbf {{u}}\Vert _2 \le C$ for some constant $C < \infty$. In addition, let

$$\begin{aligned} {\textbf{R}}_i(\mathbf {{u}}) \equiv \nabla \!f \big (\textbf{X}_i, {\varvec{\beta }}^0+m^{-1/2} \mathbf {{u}}\big ) g_\tau \big (Y_i- f(\textbf{X}_i, {\varvec{\beta }}^0+m^{-1/2} \mathbf {{u}})\big ) - \nabla \!f (\textbf{X}_i, {\varvec{\beta }}^0) g_\tau (\varepsilon _i), \end{aligned}$$

for $i=m+1, \ldots , m+T_m$, where the convergence rate of $\widehat{{\varvec{\beta }}_m}$ derived above is used. The following lemma is crucial for the proofs of the main theorems.

### Lemma 4

Let Assumptions (A1), (A2), (D), and (E) be satisfied and let the null hypothesis in (5) hold. Then, for any constants $C_1, C_2>0$ and all $k \in \mathbb {N}$ large enough, there exists a constant $C_3>0$ such that

$$\begin{aligned} \mathbb {P}\bigg [\sup _{\Vert \mathbf {{u}}\Vert _2 \le C_1} \big \Vert {\textbf{r}}_{m,k}(\mathbf {{u}}) - \mathbb {E}[{\textbf{r}}_{m,k}(\mathbf {{u}}) ] \big \Vert _1 \ge p C_2 C_3 \sqrt{\frac{k}{m}} \sqrt{ \log k}\bigg ] \le 4k^{-C_2^2} \end{aligned}$$

for $m \in \mathbb {N}$ sufficiently large.

### Proof of Lemma 4

For any observation $i \in \{m+1, \dots , m+T_m\}$, any vector $\mathbf {{u}}=(u_1, \dots , u_p) \in \mathbb {R}^p$ such that $\Vert \mathbf {{u}}\Vert _2 \le C_1$, we can express ${\textbf{R}}_i(\mathbf {{u}})$ as

A5 $$\begin{aligned} {\textbf{R}}_i(\mathbf {{u}})= & {} \nabla \!f \big (\textbf{X}_i, {\varvec{\beta }}^0+m^{-1/2} \mathbf {{u}}\big ) \Big [g_\tau \big (Y_i- f(\textbf{X}_i, {\varvec{\beta }}^0+m^{-1/2} \mathbf {{u}})\big ) -g_\tau (\varepsilon _i) \Big ] \nonumber \\{} & {} + g_\tau (\varepsilon _i) \Big [\nabla \!f \big (\textbf{X}_i, {\varvec{\beta }}^0+m^{-1/2} \mathbf {{u}}\big ) - \nabla \!f (\textbf{X}_i, {\varvec{\beta }}^0) \Big ]\equiv {\textbf {B}}^{(1)}_i(\mathbf {{u}})+{\textbf {B}}^{(2)}_i(\mathbf {{u}}). \nonumber \\ \end{aligned}$$

Both terms in (A5) will be studied separately. Let us start with ${\textbf {B}}^{(1)}_i(\mathbf {{u}})$. The function $g_\tau$ can be expressed as $g_\tau (x)=2 \tau x +2 x (1- 2 \tau ) \mathbb {I}_{\{x<0\}}$. Using the notation $v_i \equiv f(\textbf{X}_i,{\varvec{\beta }}^0) - f(\textbf{X}_i,{\varvec{\beta }}^0 + m^{-1/2} \mathbf {{u}})$ it holds with probability 1 that

A6 $$\begin{aligned}{} & {} g_\tau \Big (Y_i- f(\textbf{X}_i, {\varvec{\beta }}^0+m^{-1/2} \mathbf {{u}})\Big ) -g_\tau (\varepsilon _i)\nonumber \\ {}{} & {} \quad = 2 \tau v_i + 2(1-2 \tau ) \Big [\big (\varepsilon _i +v_i \big ) \mathbb {I}_{\{\varepsilon _i<- v_i \}} - \varepsilon _i \mathbb {I}_{\{\varepsilon _i <0\}} \Big ]. \end{aligned}$$

Let us consider a random variable $W_i \equiv \varepsilon _i \big [\mathbb {I}_{\{\varepsilon _i<- v_i \}} -\mathbb {I}_{\{\varepsilon _i <0\}} \big ]$. It holds that

$$\begin{aligned} |W_i | = |\varepsilon _i | \mathbb {I}_{\{\min (0, - v_i) \le \varepsilon _i < \max (0,- v_i) \}}, \end{aligned}$$

which also implies

A7 $$\begin{aligned} \mathbb {P}\big [|W_i | < |v_i | \big ] =1. \end{aligned}$$

The relation in (A6) can be written as

A8 $$\begin{aligned} g_\tau (\varepsilon _i +v_i) -g_\tau (\varepsilon _i) =2 \tau v_i +2 (1-2 \tau ) \big [ W_i+v_i \mathbb {I}_{\{ \varepsilon _i <-v_i\}}\big ], \end{aligned}$$

which holds with probability one. On the other hand, by Assumptions (A1) and (A2) we have that $\Vert \nabla \!f( {\textbf{x}}, {\varvec{\beta }}) \Vert _2$ is bounded for all ${\textbf{x}}\in \varUpsilon$ and all ${\varvec{\beta }}\in \mathcal{V}_m({\varvec{\beta }}^0, \mathbf {{u}})$, with $\mathcal{V}_m({\varvec{\beta }}^0,\mathbf {{u}}) = \{ {\varvec{\beta }}; \;\; \Vert {\varvec{\beta }}-{\varvec{\beta }}^0\Vert _2 \le m^{-1/2} \Vert \mathbf {{u}}\Vert _2\}$. For the right-hand side of (A8), using the fact that $\Vert \nabla \!f( {\textbf{x}}, {\varvec{\beta }}) \Vert _2$ is bounded, together with relation (A7), by applying the first order Taylor expansion to $f(\textbf{X}_i, {\varvec{\beta }}^0+m^{-1/2} \mathbf {{u}})$, we have that there exists $C>0$ such that we can write for the left-hand side of (A8)

A9 $$\begin{aligned} \mathbb {P}\big [ - C m^{-1/2}< g_\tau (\varepsilon _i+v_i) - g_\tau (\varepsilon _i) < C m^{-1/2}\big ] =1. \end{aligned}$$

Using the relations in (A6), (A8), and (A9), together with the fact that $\Vert \nabla \!f( {\textbf{x}}, {\varvec{\beta }}) \Vert _2$ is bounded for all ${\textbf{x}}\in \varUpsilon$ and ${\varvec{\beta }}\in \mathcal{V}_m({\varvec{\beta }}^0, \mathbf {{u}})$ and applying the Hoeffding inequality, we obtain that

$$\begin{aligned} \mathbb {P}\left[ \bigg | \sum ^{m+k}_{i=m+1} \frac{\partial f}{\partial \beta _j}(\textbf{X}_i, {\varvec{\beta }}^0+m^{-1/2} \mathbf {{u}}) G_\tau (\varepsilon _i, v_i) \bigg | \ge t\right] \le 2 \exp \bigg (- \frac{2 t^2}{4 C^2 k/m} \bigg ), \end{aligned}$$

for all $t \in \mathbb {R}$ and $j=1, \dots , p$, where, for brevity, use used the notation

$$\begin{aligned} G_\tau (\varepsilon _i, v_i) = \big [g_\tau (\varepsilon _i - v_i) - g_\tau (\varepsilon _i) \big ] - \mathbb {E}\big [g_\tau (\varepsilon _i - v_i) - g_\tau (\varepsilon _i) \big ]. \end{aligned}$$

Next, similarly as in the proof of Lemma 1 in Ciuperca (2022), under Assumptions (A1) and (A2), using the last relation above, we have that for all constants $C_1, C_4 >0$, there exists a constant $\widetilde{C}>0$ such that

A10 $$\begin{aligned}&\mathbb {P}\left[ \bigg \Vert \sum ^{m+k}_{i=m+1} \big ({\textbf {B}}^{(1)}_i(\mathbf {{u}}) -\mathbb {E}[{\textbf {B}}^{(1)}_i(\mathbf {{u}})] \big )\bigg \Vert _1 \ge \sqrt{2} C_4 \widetilde{C} p \sqrt{\frac{k}{m}} \sqrt{\log k}\right] \nonumber \\&\quad \le \mathbb {P}\left[ \max _{1 \leqslant j \leqslant p} \bigg | \sum ^{m+k}_{i=m+1}\frac{\partial f}{\partial \beta _j}(\textbf{X}_i, {\varvec{\beta }}^0+m^{-1/2} \mathbf {{u}})G_\tau (\varepsilon _i, v_i) \bigg | \ge \sqrt{2} C_4 \widetilde{C} \sqrt{\frac{k}{m}} \sqrt{\log k} \right] \nonumber \\&\quad \le 2 k^{-C_4^2}. \end{aligned}$$

Next, we proceed by studying the random vector ${\textbf {B}}^{(2)}_i(\mathbf {{u}})$ from the relation in (A5). Let us denote its *j*-th elemen, for $j=1, \dots , p$, as

$$\begin{aligned} B^{(2)}_{ij}(\mathbf {{u}})&= \left[ \frac{\partial f}{\partial \beta _j}(\textbf{X}_i , {\varvec{\beta }}^0+m^{-1/2} \mathbf {{u}}) - \frac{\partial f}{\partial \beta _j}(\textbf{X}_i , {\varvec{\beta }}^0) \right] g_\tau (\varepsilon _i)\\&= m^{-1/2} \mathbf {{u}}\nabla ^2_j f(\textbf{X}_i , {\varvec{\beta }}^0+m^{-1/2} \theta _{ji} \mathbf {{u}}) g_\tau (\varepsilon _i), \end{aligned}$$

for some constants $\theta _{ji} \in [0,1]$. Under Assumption (D), it holds that $\mathbb {V}\text{ ar }\,[g_\tau (\varepsilon _i)] < \infty$ and also $\mathbb {E}[g^2_\tau (\varepsilon _i)]< C < \infty$. Using these two relations, we obtain that

$$\begin{aligned} \mathbb {V}\text{ ar }\,[B_{ij}^{(2)}(\mathbf {{u}})] \le \mathbb {E}[\big (B^{(2)}_{ij}(\mathbf {{u}})\big )^2] \le C m^{-1} \Vert \mathbf {{u}}\Vert _2^2 \Vert \nabla ^2_jf (\textbf{X}_i, {\varvec{\beta }}^0 +m^{-1/2} \theta _{ji} \mathbf {{u}}_i) \Vert ^2_2, \end{aligned}$$

for each $j \in \{ 1, \dots , p\}$, where the Cauchy–Schwarz inequality was applied in the last step. Moreover, using Assumption (E) we have that for all $C_1>0$ there exists a constant $C_5>0$ such that

$$\begin{aligned} \max _{m+1 \leqslant i \leqslant m+k} \left( \max _{\Vert \mathbf {{u}}\Vert _2 \le C_1}\Vert \nabla ^2_j f (\textbf{X}_i, {\varvec{\beta }}^0 +m^{-1/2} \mathbf {{u}}) \Vert _2 \right) \le C_5. \end{aligned}$$

Therefore, we also obtain that

A11 $$\begin{aligned} \sum ^{m+k}_{i=m+1} \mathbb {V}\text{ ar }\,[B^{(2)}_{ij}(\mathbf {{u}})] =O(k/m). \end{aligned}$$

For ${\textbf {B}}^{(2)}_i(\mathbf {{u}})$, taking into account the relation in (A11) and the fact that $B^{(2)}_{ij}(\mathbf {{u}})$ is uniformly bounded by $C m^{-1/2}$, we can use Lemma 4.1 of Ciuperca (2017) for $\delta _k =k/m$. Then, for $k \in \mathbb {N}$ being sufficiently large, for any constant $C_6>0$ and $\Vert \mathbf {{u}}\Vert _2 <C_1$, we have

$$\begin{aligned} \mathbb {P}\left[ \bigg | \sum ^{m+k}_{i=m+1} \big [B^{(2)}_{ij}(\mathbf {{u}}) - \mathbb {E}[ B^{(2)}_{ij}(\mathbf {{u}})]\big ] \bigg | \ge (1+C_6) \sqrt{\frac{k}{m}} \sqrt{\log k} \right] \le 2 k^{-C_6}, \end{aligned}$$

which further implies, similarly as for (A10), that

A12 $$\begin{aligned} \mathbb {P}\left[ \Big \Vert \sum ^{m+k}_{i=m+1} [{\textbf {B}}^{(2)}_{i}(\mathbf {{u}}) - \mathbb {E}[ {\textbf {B}}^{(2)}_{i}(\mathbf {{u}})]]\Big \Vert _1 \ge p (1+C_6)\sqrt{\frac{k}{m}} \sqrt{\log k} \right] \le 2 k^{-C_6}. \end{aligned}$$

Taking now $C_6=C_4^2$ and $\widetilde{\widetilde{C}}=2^{-1/2} (1+C_4^2)/C_4$ we get

A13 $$\begin{aligned} \mathbb {P}\left[ \Big \Vert \sum ^{m+k}_{i=m+1} [{\textbf {B}}^{(2)}_{i}(\mathbf {{u}}) - \mathbb {E}[ {\textbf {B}}^{(2)}_{i}(\mathbf {{u}})]]\Big \Vert _1 \ge \sqrt{2} p \widetilde{\widetilde{C}} C_4 \sqrt{\frac{k}{m}} \sqrt{\log k} \right] \le 2 k^{-C_4^2}. \end{aligned}$$

Moreover, for any constant $c > 0$ and any two random vectors ${\textbf {V}}_1$ and ${\textbf {V}}_2$ of the same size, it holds that

A14 $$\begin{aligned} \begin{aligned} \mathbb {P}[\Vert {\textbf {V}}_1+{\textbf {V}}_2 \Vert _1 \ge c]&\le \mathbb {P}[\Vert {\textbf {V}}_1 \Vert _1 +\Vert {\textbf {V}}_2\Vert _1 \ge c ] \le \mathbb {P}\bigg [ \bigg ( \Vert {\textbf {V}}_1\Vert _1 \ge \frac{c}{2}\bigg ) \cup \bigg ( \Vert {\textbf {V}}_2\Vert _1 \ge \frac{c}{2}\bigg )\bigg ] \\&\le \mathbb {P}\bigg [ \Vert {\textbf {V}}_1\Vert _1 \ge \frac{c}{2}\bigg ] +\mathbb {P}\bigg [ \Vert {\textbf {V}}_2\Vert _1 \ge \frac{c}{2}\bigg ]. \end{aligned} \end{aligned}$$

For the first inequality in (A14) we used the fact that for any constant $c>0$ the event $c \le \Vert {\textbf {V}}_1+{\textbf {V}}_2 \Vert _1 \le \Vert {\textbf {V}}_1 \Vert _1 +\Vert {\textbf {V}}_2\Vert _1$ implies, with probability one, also the random event

$$\begin{aligned} \big (\Vert {\textbf {V}}_1+{\textbf {V}}_2 \Vert _1 \ge c \big ) \subseteq \big (\Vert {\textbf {V}}_1 \Vert _1 +\Vert {\textbf {V}}_2\Vert _1 \ge c \big ) \subseteq \bigg ( \max \big (\Vert {\textbf {V}}_1\Vert _1,\Vert {\textbf {V}}_2\Vert _1 \big ) \ge c/2\bigg ), \end{aligned}$$

where we considered ${\textbf {V}}_1={\textbf {B}}^{(1)}_i(\mathbf {{u}}) - \mathbb {E}[{\textbf {B}}^{(1)}_i(\mathbf {{u}})]$ and ${\textbf {V}}_2={\textbf {B}}^{(2)}_i(\mathbf {{u}}) - \mathbb {E}[{\textbf {B}}^{(2)}_i(\mathbf {{u}})]$. Taking now the relations in (A10), (A13) and (A14), and the constants $C_4=C_2$, $C_3=2^{-3/2}\min (\widetilde{C},\widetilde{\widetilde{C}})$, and $c=p C_2 C_3 (k / m)^{1/2} (\log k)^{1/2}$, we obtain that

$$\begin{aligned} \mathbb {P}\left[ \sum ^{m+k}_{i=m+1} \big \Vert {\textbf {R}}_{i}(\mathbf {{u}}) - \mathbb {E}[ {\textbf {R}}_{i}(\mathbf {{u}})]\big \Vert _1 \ge p C_2 C_3 \sqrt{\frac{k}{m}} \sqrt{\log k} \right] \le 4 k^{-C_2^2}, \end{aligned}$$

for all $C_2>0$, which completes the proof of the lemma. $\square$

### Proof of Theorem 2

If *k* is fixed then $z(m,k,\gamma ) {\underset{m \rightarrow \infty }{\longrightarrow }} \infty$. With respect to Assumptions (A1), (A2), (B), and (D), we have that

$$\begin{aligned} \left\| \textbf{J}^{-1/2}_m (\widehat{\varvec{\beta }}_m) \sum ^{m+k}_{i=m+1} \nabla \!f(\textbf{X}_i, \widehat{\varvec{\beta }}_m) g_\tau (\widehat{\varepsilon }_i)\right\| _\infty \overset{{\mathbb {P}}}{\underset{m \rightarrow \infty }{\longrightarrow }} 0. \end{aligned}$$

Therefore, in this case, we also obtain that

$$\begin{aligned} \frac{\Vert \mathbf{{S}}(m,k) \Vert _\infty }{z(m,k,\gamma )} \overset{\mathbb {P}}{\underset{m \rightarrow \infty }{\longrightarrow }} 0. \end{aligned}$$

If $k \rightarrow \infty$ (i.e., $k \equiv k_m \rightarrow \infty$ for $m \rightarrow \infty$), then by Lemma 4 we have for $\mathbf {{u}}\in \mathbb {R}^p$, with $\Vert \mathbf {{u}}\Vert _1 \le C_1$: ${\textbf{r}}_{m,k}(\mathbf {{u}})=\mathbb {E}[{\textbf{r}}_{m,k}(\mathbf {{u}})]+O_\mathbb {P}\big (m^{-1/2} k^{1/2} (\log k)^{1/2}\big )$.

On the other hand, for $\mathbb {E}[{\textbf{r}}_{m,k}(\mathbf {{u}})]$ we have, using Assumptions (A1), (A2), (B), (D), and (E), that

$$\begin{aligned}&\mathbb {E}[{\textbf{r}}_{m,k}(\mathbf {{u}})] \\&\quad = \sum ^{m+k}_{i=m+1} \nabla \!f(\textbf{X}_i, {\varvec{\beta }}^0+m^{-1/2} \mathbf {{u}})\mathbb {E}\Big [ g_\tau (\varepsilon _i) - h_\tau (\varepsilon _i) \big (f(\textbf{X}_i, {\varvec{\beta }}^0+m^{-1/2} \mathbf {{u}}) - f(\textbf{X}_i, {\varvec{\beta }}^0)\big )\\&\qquad +o_\mathbb {P}\big (f(\textbf{X}_i, {\varvec{\beta }}^0+m^{-1/2} \mathbf {{u}}) - f(\textbf{X}_i, {\varvec{\beta }}^0)\big ) \Big ] \\&\quad =- \sum ^{m+k}_{i=m+1} \Big [\nabla \!f(\textbf{X}_i, {\varvec{\beta }}^0+m^{-1/2} \mathbf {{u}}) m^{-1/2} \nabla \!f^\top (\textbf{X}_i, {\varvec{\beta }}^0) \mathbf {{u}}\mathbb {E}[h_\tau (\varepsilon )]+o_\mathbb {P}(m^{-1/2}) \Big ] \\&\quad =- \mathbb {E}[h_\tau (\varepsilon )] ~ m^{-1/2}\sum ^{m+k}_{i=m+1} \nabla \!f(\textbf{X}_i, {\varvec{\beta }}^0) \nabla \!f^\top (\textbf{X}_i, {\varvec{\beta }}^0) \mathbf {{u}}+o(k m^{-1/2}). \end{aligned}$$

Thus, we also have that

A15 $$\begin{aligned} {\textbf{r}}_{m,k}(\mathbf {{u}}) =&- m^{-1/2} \mathbb {E}[h_\tau (\varepsilon )] \sum ^{m+k}_{i=m+1} \nabla \!f(\textbf{X}_i, {\varvec{\beta }}^0)\nabla \!f^\top (\textbf{X}_i, {\varvec{\beta }}^0) \mathbf {{u}}\nonumber \\&+O_\mathbb {P}(m^{-1/2} k^{1/2} (\log k)^{1/2}). \end{aligned}$$

Using the relation in (A3) and taking $\mathbf {{u}}=m^{1/2} (\widehat{\varvec{\beta }}_m -{\varvec{\beta }}^0)$ in the relation in (A15), we obtain

$$\begin{aligned} {\textbf{r}}_{m,k}(m^{1/2} (\widehat{\varvec{\beta }}_m -{\varvec{\beta }}^0))&= -m^{-1} \textbf{V}^{-1}_m({\varvec{\beta }}^0) \left( \sum ^{m+k}_{i=m+1} \nabla \!f(\textbf{X}_i, {\varvec{\beta }}^0)\nabla \!f^\top (\textbf{X}_i, {\varvec{\beta }}^0) \right) \\&\quad \times \left( \sum ^m_{i=1} \nabla \!f (\textbf{X}_i,{\varvec{\beta }}^0) g_\tau (\varepsilon _i)+o_\mathbb {P}(m^{1/2})\right) \\&\quad + O_\mathbb {P}\Big (m^{-1/2} k^{1/2} (\log k)^{1/2}\Big ) \end{aligned}$$

and, using Assumption (C), also

$$\begin{aligned} {\textbf{r}}_{m,k}(m^{1/2} (\widehat{\varvec{\beta }}_m -{\varvec{\beta }}^0)) =&-k m^{-1}\left( \sum ^m_{i=1} \nabla \!f (\textbf{X}_i,{\varvec{\beta }}^0) g_\tau (\varepsilon _i)+o_\mathbb {P}(m^{1/2})\right) \\&+O_\mathbb {P}(m^{-1/2} k^{1/2} (\log k)^{1/2}). \end{aligned}$$

On the other hand, by the definition of the random process ${\textbf{r}}_{m,k}$, we get, with probability one, that

$$\begin{aligned} {\textbf{r}}_{m,k}(m^{1/2}(\widehat{\varvec{\beta }}_m -{\varvec{\beta }}^0)) =\sum ^{m+k}_{i=m+1}\big [ \nabla \!f (\textbf{X}_i, \widehat{\varvec{\beta }}_m) g_\tau (\widehat{\varepsilon }_i) - \nabla \!f (\textbf{X}_i,{\varvec{\beta }}^0) g_\tau (\varepsilon _i) \big ]. \end{aligned}$$

The last two relations imply

A16 $$\begin{aligned} \sum ^{m+k}_{i=m+1}\nabla \!f (\textbf{X}_i, \widehat{\varvec{\beta }}_m) g_\tau (\widehat{\varepsilon }_i)= & {} \sum ^{m+k}_{i=m+1} \nabla \!f (\textbf{X}_i,{\varvec{\beta }}^0) g_\tau (\varepsilon _i) -k m^{-1} \sum ^m_{i=1} \nabla \!f (\textbf{X}_i,{\varvec{\beta }}^0) g_\tau (\varepsilon _i)\nonumber \\{} & {} + o_\mathbb {P}(k m^{-1/2})+O_\mathbb {P}(m^{-1/2} k^{1/2} (\log k)^{1/2}). \end{aligned}$$

The rest of the proof follows the same lines as the proof of Theorem 1 in Ciuperca (2022) using the Komlós–Major–Tusnády (KMT) approximation for independent random vectors and Theorem 2.1 of Horváth et al (2004). Let us only sketch the main idea of the end of the proof. By the KMT approximation for independent random variables not identically distributed (see Götze and Zaitsev (2009)) for each component of the random vectors $\big (\textbf{J}^{-1/2}_m ({\varvec{\beta }}^0) \nabla \!f (\textbf{X}_i,{\varvec{\beta }}^0) g_\tau (\varepsilon _i) \big )_{1 \leqslant i \leqslant m}$ and of $\big (\textbf{J}^{-1/2}_m ({\varvec{\beta }}^0) \nabla \!f (\textbf{X}_i,{\varvec{\beta }}^0) g_\tau (\varepsilon _i) \big )_{m+1 \leqslant i \leqslant m+T_m}$, we have that for all $\nu >3$ and $m \rightarrow \infty$, there exists two Wiener processes $\big \{{\textbf {W}}_{1,m}(t), t \in [0,\infty )\big \}$ and $\big \{{\textbf {W}}_{2,m}(t), t \in [0, \infty ) \big \}$ of the dimension *p* such that for the two terms of the right hand side of the relation in (A16) it holds

A17 $$\begin{aligned} \sup _{1\leqslant k < \infty } k^{-1/\nu } \Big \Vert \textbf{J}^{-1/2}_m ({\varvec{\beta }}^0) \sum ^{m+k}_{i=m+1}\nabla \!f (\textbf{X}_i,{\varvec{\beta }}^0) g_\tau (\varepsilon _i) - {\textbf {W}}_{1,m}(k) \Big \Vert _\infty =O_\mathbb {P}(1) \end{aligned}$$

and

A18 $$\begin{aligned} \Big \Vert \textbf{J}^{-1/2}_m ({\varvec{\beta }}^0) \sum ^m_{i=1} \nabla \!f (\textbf{X}_i,{\varvec{\beta }}^0) g_\tau (\varepsilon _i) - {\textbf {W}}_{2,m}(m) \Big \Vert _\infty =o_\mathbb {P}(m^{1/\nu }). \end{aligned}$$

By the proof of Theorem 2.1 of Horváth et al (2004), for any $T>0$, we have

A19 $$\begin{aligned} \sup _{1\leqslant k < \infty }&\frac{\big \Vert {\textbf {W}}_{1,m}(k) - \frac{k}{m}{} {\textbf {W}}_{2,m}(m)\big \Vert _\infty }{z(m,k,\gamma )} \nonumber \\&\overset{\mathcal{L}}{\underset{m \rightarrow \infty }{=}} \max _{1 \leqslant k \leqslant mT} \sqrt{m}\frac{\big \Vert W_1(k/m)- (k/m) W_2(1)\big \Vert _\infty }{z(m,k,\gamma )} \end{aligned}$$

and also

A20 $$\begin{aligned} \max _{1 \leqslant k \leqslant mT} \sqrt{m}\frac{\big \Vert W_1(k/m)- (k/m) W_2(1)\big \Vert _\infty }{z(m,k,\gamma )} \overset{\mathcal{L}}{\underset{m \rightarrow \infty }{\longrightarrow }} \sup _{0< t < L(T) } \frac{\Vert {\textbf {W}}_p(t)\Vert _\infty }{t^\gamma }. \end{aligned}$$

On the other hand, by Assumptions (C) and (D), together with Proposition 1, we have that $\textbf{J}^{-1/2}_m (\widehat{\varvec{\beta }}_m)$ converges to $\textbf{J}^{-1/2}_m ({\varvec{\beta }}^0)$ with the convergence rate of the order $m^{-1/2}$. Thus, $\textbf{J}^{-1/2}_m (\widehat{\varvec{\beta }}_m) = \textbf{J}^{-1/2}_m ({\varvec{\beta }}^0) +O_\mathbb {P}(m^{-1/2})$. The proof of the theorem now follows by combining this last relation together with the relations in (A16), (A17), (A18), and (A19). $\square$

### Proof of Theorem 3

It will be shown that there exists an observation with an index $\widetilde{k}_m \in \{m + 1, \dots , m + T_m\}$ for which $\Vert \mathbf{{S}}(m,\widetilde{k}_m) \Vert _\infty / z(m,\widetilde{k}_m,\gamma ) \overset{\mathbb {P}}{\underset{m \rightarrow \infty }{\longrightarrow }} \infty .$

We consider the open-end procedure case. Let us consider $\widetilde{k}_m=k^0_m+m^s$, with $s>1$. Since the function $(x+1)(x/(1+x))^{-\gamma }$ is increasing in $x>0$ for $\gamma \in [0,1/2)$, we have, as in the proof of Theorem 1, that there exists $C>0$ such that,

A21 $$\begin{aligned} \frac{\Vert \textbf{J}_m^{-1/2}(\widehat{\varvec{\beta }}_m) \sum ^{m+k^0_m}_{i=m+1}\nabla \!f (\textbf{X}_i, \widehat{\varvec{\beta }}_m) g_\tau \big (\varepsilon _i +f(\textbf{X}_i ,{\varvec{\beta }}^0) - f(\textbf{X}_i, \widehat{\varvec{\beta }}_m)\big ) \Vert _\infty }{z(m, \widetilde{k}_m, \gamma )}<C < \infty , \end{aligned}$$

with probability converging to 1, when $m \rightarrow \infty$.

It remains to study $\sum ^{m+\widetilde{k}_m}_{i=m+k^0_m+1} \nabla \!f (\textbf{X}_i, \widehat{\varvec{\beta }}_m) g_\tau \big (\varepsilon _i +f(\textbf{X}_i,{\varvec{\beta }}^1) - f(\textbf{X}_i, \widehat{\varvec{\beta }}_m)\big )$ or more precisely, taking into account the convergence rate of $\widehat{\varvec{\beta }}_m$, we are going to study

$$\begin{aligned} \sum ^{m+\widetilde{k}_m}_{i=m+k^0_m+1} \nabla \!f (\textbf{X}_i, {\varvec{\beta }}^0+m^{-1/2} \mathbf {{u}}) g_\tau \big (\varepsilon _i +f(\textbf{X}_i,{\varvec{\beta }}^1) - f(\textbf{X}_i, {\varvec{\beta }}^0+m^{-1/2} \mathbf {{u}})\big ) \end{aligned}$$

with $\mathbf {{u}}\in \mathbb {R}^p$, $\Vert \mathbf {{u}}\Vert _2 <C$. Consider the following sum

A22 $$\begin{aligned} \sum ^{m+\widetilde{k}_m}_{i=m+k^0_m+1} {\textbf {R}}_{i}(\mathbf {{u}}) =&- \sum ^{m+\widetilde{k}_m}_{i=m+k^0_m+1} \nabla \!f(\textbf{X}_i, {\varvec{\beta }}^0) g_\tau (\varepsilon _i)\nonumber \\&\quad + \sum ^{m+\widetilde{k}_m}_{i=m+k^0_m+1} \Big [ \nabla \!f (\textbf{X}_i, {\varvec{\beta }}^0+m^{-1/2} \mathbf {{u}}) g_\tau \big (\varepsilon _i \nonumber \\&\quad +f(\textbf{X}_i ,{\varvec{\beta }}^1) - f(\textbf{X}_i, {\varvec{\beta }}^0+m^{-1/2} \mathbf {{u}})\big )\Big ]. \end{aligned}$$

Then, taking into account Assumption (D), the expectation $\mathbb {E}\left[ \sum ^{m+\widetilde{k}_m}_{i=m+k^0_m+1} {\textbf {R}}_{i}(\mathbf {{u}})\right]$ on the left hand-side of the equation in (A22) equals to

$$\begin{aligned} \sum ^{m+\widetilde{k}_m}_{i=m+k^0_m+1} \nabla \!f (\textbf{X}_i, {\varvec{\beta }}^0+m^{-1/2} \mathbf {{u}}) \mathbb {E}\big [ g_\tau \big (\varepsilon _i +f(\textbf{X}_i +{\varvec{\beta }}^1) - f(\textbf{X}_i, {\varvec{\beta }}^0+m^{-1/2} \mathbf {{u}})\big ) \big ]. \end{aligned}$$

Therefore, it remains to study $\mathbb {E}\big [ g_\tau \big (\varepsilon _i +f(\textbf{X}_i +{\varvec{\beta }}^1) - f(\textbf{X}_i, {\varvec{\beta }}^0+m^{-1/2} \mathbf {{u}})\big ) \big ]=\mathbb {E}\big [ g_\tau \big (\varepsilon _i +f(\textbf{X}_i +{\varvec{\beta }}^1) - f(\textbf{X}_i, {\varvec{\beta }}^0+m^{-1/2} \mathbf {{u}})\big )- g_\tau (\varepsilon _i) \big ]$, where Assumption (D) was applied. By Lemma 2 of Gu and Zou (2016), we have that there exists a constant $c_i$, with $|c_i| \in [2\underline{c}, 2 \bar{c}]$, such that $g_\tau \big (\varepsilon _i +f(\textbf{X}_i +{\varvec{\beta }}^1) - f(\textbf{X}_i, {\varvec{\beta }}^0+m^{-1/2} \mathbf {{u}})\big )- g_\tau (\varepsilon _i) =c_i \big [f(\textbf{X}_i, {\varvec{\beta }}^1) - f(\textbf{X}_i, {\varvec{\beta }}^0+m^{-1/2} \mathbf {{u}}) \big ]$, with probability one. Thus, we obtain

$$\begin{aligned} \frac{1}{\widetilde{k}_m - k^0_m}&\left\| \sum ^{m+\widetilde{k}_m}_{i=m+k^0_m+1} \mathbb {E}[{\textbf {R}}_{i}(\mathbf {{u}})]\right\| _\infty \\&= \frac{1}{\widetilde{k}_m - k^0_m} \left\| \sum ^{m+\widetilde{k}_m}_{i=m+k^0_m+1} \frac{c_i \nabla \!f(\textbf{X}_i, {\varvec{\beta }}^0+m^{-1/2} \mathbf {{u}})}{\big [ f(\textbf{X}_i, {\varvec{\beta }}^1) - f(\textbf{X}_i, {\varvec{\beta }}^0+m^{-1/2} \mathbf {{u}})\big ]^{-1}}\right\| _\infty . \end{aligned}$$

Taking into account this last relation, taking into account the fact that for all $c_i$ such that $|c_i| \in [2\underline{c}, 2 \bar{c}]$ there exists some constant $C>0$ such that

$$\begin{aligned} \frac{1}{m^s} \left\| \sum ^{m+\widetilde{k}_m}_{i=m+k^0_m+1} c_i \nabla \!f(\textbf{X}_i, {\varvec{\beta }}^0) \big [ f(\textbf{X}_i, {\varvec{\beta }}^1) - f(\textbf{X}_i, {\varvec{\beta }}^0)\big ]\right\| _\infty>C>0, \end{aligned}$$

using the Kolmogorov strong Law of Large Numbers for independent not identically distributed random variables $R_{ij}(\mathbf {{u}})$, we finally obtain that

A23 $$\begin{aligned} \left\| \sum ^{m+\widetilde{k}_m}_{i=m+k^0_m+1} {\textbf {R}}_{i}(\mathbf {{u}})\right\| _\infty =O_\mathbb {P}\big ( (\widetilde{k}_m - k^0_m) \Vert {\varvec{\beta }}^1-{\varvec{\beta }}^0 \Vert _2\big ). \end{aligned}$$

On the other hand, by the Central Limit Theorem, we also have

A24 $$\begin{aligned} \sum ^{m+\widetilde{k}_m}_{i=m+k^0_m+1} \nabla \!f(\textbf{X}_i, {\varvec{\beta }}^0) g_\tau (\varepsilon _i) =O_\mathbb {P}(\widetilde{k}_m - k^0_m)^{1/2} =O_\mathbb {P}(m^{s/2}). \end{aligned}$$

Therefore, the relations in (A22), (A23), and (A24) together imply

$$\begin{aligned} \frac{\big \Vert \textbf{J}_m^{-1/2}({\varvec{\beta }}^0+\frac{\mathbf {{u}}}{\sqrt{m}})\sum ^{m+\widetilde{k}_m}_{i=m+k^0_m+1} \mathcal {N}_i \big \Vert _\infty }{z(m,\widetilde{k}_m,\gamma )} = \frac{O_\mathbb {P}(m^{s/2}) +O_\mathbb {P}\big ( m^s \Vert {\varvec{\beta }}^1 - {\varvec{\beta }}^0 \Vert _2\big )}{z(m,\widetilde{k}_m,\gamma )}, \end{aligned}$$

where, again for brevity, $\mathcal {N}_i$ is defined as

$$\begin{aligned} \mathcal {N}_i = \nabla \!f\Big (\textbf{X}_i, {\varvec{\beta }}^0+ \frac{\mathbf {{u}}}{\sqrt{m}}\Big ) g_\tau \left( \varepsilon _i +f(\textbf{X}_i +{\varvec{\beta }}^1)\! -\! f\left( \textbf{X}_i, {\varvec{\beta }}^0+ \frac{\mathbf {{u}}}{\sqrt{m}}\right) \right) . \end{aligned}$$

Moreover, it also hold that

A25 $$\begin{aligned} \frac{O_\mathbb {P}(m^{s/2}) +O_\mathbb {P}\big ( m^s \Vert {\varvec{\beta }}^1 - {\varvec{\beta }}^0 \Vert _2\big )}{z(m,\widetilde{k}_m,\gamma )} {\underset{m \rightarrow \infty }{\longrightarrow }} \infty . \end{aligned}$$

Since $\Vert \textbf{J}_m^{-1/2}({\varvec{\beta }}^0+m^{-1/2} \mathbf {{u}}) -\textbf{J}_m^{-1/2}({\varvec{\beta }}^0) \Vert _2 {\underset{m \rightarrow \infty }{\longrightarrow }} 0$, where $\Vert . \Vert _2$ is the spectral norm, the theorem follows by relations in (A21) and (A25). $\square$

## References

[CR1] Aue A, Horváth L, Reimherr M (2009). Delay times of sequential procedures for multiple time series regression models. J Economet.

[CR2] Barassi M, Horváth L, Zhao Y (2020). Change-point detection in the conditional correlation structure of multivariate volatility models. J Bus Econom Stat.

[CR3] Basseville M, Nikiforov I (1993). Detection of abrupt changes: theory and applications.

[CR4] Bellini F, Bignozzi V, Puccetti G (2018). Conditional expectiles, time consistency and mixture convexity properties. Insur Math Econ.

[CR5] Chambers J (1973). Fitting nonlinear models: numerical techniques. Biometrika.

[CR6] Chen D, Chen X, Chen J (2020). Reconstructing and forecasting the covid-19 epidemic in the united states using a 5-parameter logistic growth model. Global Health Res Policy.

[CR7] Choi S, Kim H, Lee Y (2003). Nonlinear asymmetric least squares estimators. J Korean Statistical Soc.

[CR8] Chu CSJ, Stinchcombe M, White H (1996). Monitoring structural change. Econometrica.

[CR9] Ciuperca G (2013). Two tests for sequential detection of a change-point in a nonlinear model. J Stat Plan Infer.

[CR10] Ciuperca G (2017). Real time change-point detection in a nonlinear quantile model. Seq Anal.

[CR11] Ciuperca G (2022) Real-time detection of a change-point in a linear expectile model. Statistical Papers (to appear)

[CR12] Fremdt S (2015). Page’s sequential procedure for change-point detection in time series regression. Statistics.

[CR13] Götze F, Zaitsev A (2009). Bounds for the rate of strong approximation in the multidimensional invariance principle. Theory Probab Appl.

[CR14] Gu Y, Zou H (2016). High-dimensional generalizations of asymmetric least squares regression and their applications. Ann Stat.

[CR15] Hoga Y (2017). Monitoring multivariate time series. J Multivar Anal.

[CR16] Horváth L, Hušková M, Kokoszka P (2004). Monitoring changes in linear models. J Stat Plan Infer.

[CR17] Hušková M, Prášková Z, Steinebach J (2007). On the detection of changes in autoregressive time series. J Stat Plan Infer.

[CR18] Kim M, Lee S (2016). Nonlinear expectile regression with application to value-at-risk and expected shortfall estimation. Comput Stat Data Anal.

[CR19] Lee Y, Lee S (2019). CUSUM test for general nonlinear integer-valued GARCH models: comparison study. Ann Inst Stat Math.

[CR20] Nedényi F (2018). An online change detection test for parametric discrete-time stochastic processes. Seq Anal.

[CR21] Newey W, Powell J (1987). Asymmetric least squares estimation and testing. Econometrica.

[CR22] Philipps C (2022) Interpreting expectiles. SSRN, pp 1–39. 10.2139/ssrn.3881402

[CR23] Seber G, Wild C (2003). Nonlinear regression.

[CR24] Zhou M, Wang H, Tang Y (2015). Sequential change point detection in linear quantile regression models. Statist Probab Lett.

[CR25] Ziegel J (2016). Coherence and elicitability. Math Financ.

